# Tunable wetting properties of poly(3-hexylthiophene) films with different doping levels

**DOI:** 10.1140/epje/s10189-026-00608-5

**Published:** 2026-07-17

**Authors:** Junqi Lu, David Neusser, David Moser, Pedro M. Resende, Christopher R. McNeill, Sabine Ludwigs

**Affiliations:** 1 IPOC – Functional Polymers, Institute of Polymer Chemistry (IPOC), University of Stuttgart, 70569 Stuttgart, Germany; 2 Department of Materials Science and Engineering, Monash University, Wellington Road, Clayton, VIC 3800 Australia

## Abstract

This work systematically investigates the wetting properties of electrochemically doped poly(3-hexylthiophene) (P3HT) films at different oxidation states. In situ spectroelectrochemistry and ex situ opto-electronic characterization of the doped films confirm that the polaron and bipolaron states have successfully been obtained through electrochemical doping. Sessile drop and dynamic water contact angle measurements yield reproducible results that strongly depend on the applied electrochemical doping potential, with a maximum water contact angle variation of up to 43° from the neutral to the fully doped state. The evolution of wettability correlates with the doping states and is heavily influenced by morphological changes upon doping. Atomic force microscopy (AFM) and scanning electron microscopy (SEM) reveal that wrinkle-like structures appear at high doping levels which is consistent with predominant bipolaron formation accompanied by doping-induced swelling followed by drying. Complementary grazing-incidence wide-angle X-ray scattering (GIWAXS) shows that doping to the polaronic state leads to lamellar expansion with a contraction of the *π*–*π* stacking distance within crystalline domains. Despite these lattice changes, the edge-on orientation is largely preserved, indicating that the overall crystalline texture remains stable even at the highest studied doping levels.

**Graphical abstract:**

**Figure:**
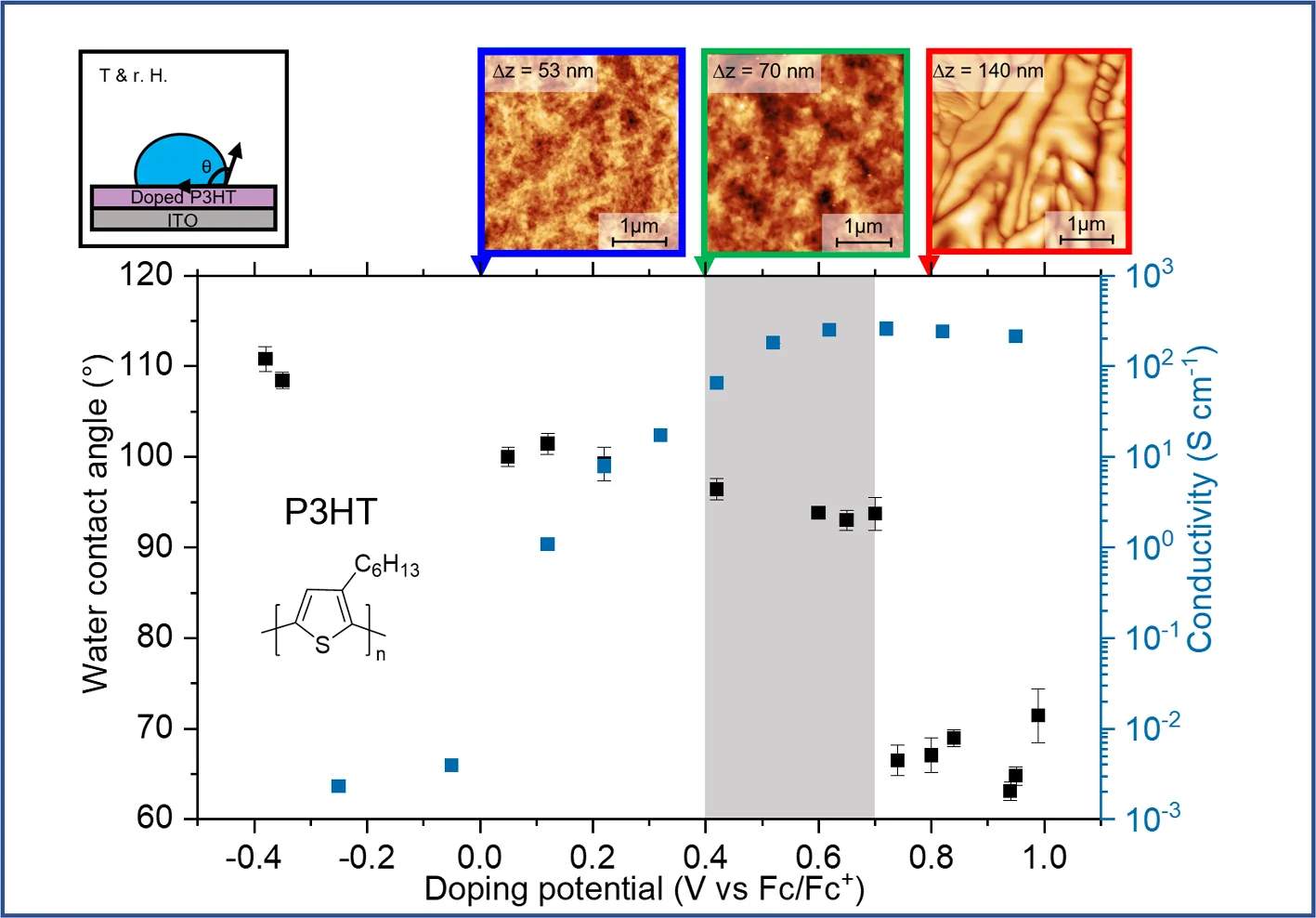
Electrochemically doped films show large differences in wettability due to surface morphology changes upon strong doping, while conductivity values remain high.

**Supplementary Information:**

The online version contains supplementary material available at 10.1140/epje/s10189-026-00608-5.

## Introduction

Owing to their inherent flexibility and metallic-level high conductivity, conducting polymers have consistently been regarded as attractive materials since their discovery in the 1970s [1, 2]. Consequently, various organic electronics applications based on conducting polymers are being explored, leveraging their unique advantages, such as lightweight nature, low working potential, and facile fabrication [3]. Conjugated polymers such as poly(3-hexylthiophene) (P3HT) with alternating single and double bonds form an extensive *π*-conjugated system, which facilitates inter- and intrachain charge transport. The key advantage of conducting polymers is their inherent tunability. Small modifications to their chemical structure, achievable through molecular design, can lead to significant changes in their band gap and thus their electrical and optical properties [4].

Our research focuses on P3HT due to its facile solution-based fabrication and its semicrystalline character, which allows to target different degrees of crystallinity depending on the processing conditions [5–8]. P3HT functions as a semiconductor, i.e., it is non-conducting in its neutral state, becoming highly conductive in its oxidized states, known as polaron and bipolaron states [9–13]. These new charge carriers create new energy levels within the band gap, thus enhancing electrical conductivity. Modification of the doping level induces significant alterations in their macroscopic functional properties, thereby enabling a wide range of applications, from organic field effect transistors (OFETs) [14], organic photovoltaics (OPVs) [15], thermoelectrics [16] and solid-state switchable devices [17–19], depending on their oxidation state.

Among these, the modulation of wetting properties, based on surface energy changes during this process, presents a particularly promising direction. Compared to the high potentials required for electrowetting, films based on conducting polymers, possessing tunable wetting properties at low potentials [20], demonstrate broader applications in microfluidics [17], self-cleaning [21], and printing technologies [22]. The wetting transition of P3HT between the neutral and polaronic states in solid-state devices has been previously investigated [23, 24]. One study focused, for example, on the wetting behavior of P3HT films electrochemically doped with different aqueous electrolyte salts [25]. Systematic studies covering higher oxidation states and their correlation to the resulting wetting properties are still lacking, particularly regarding the influence of bipolaronic species formation at high doping levels. A key challenge remains the preparation of stable, highly doped solid-state P3HT films that are suitable for reliable water contact angle measurements.

Doping of conducting polymers can be achieved through chemical or electrochemical methods. For the commonly employed chemical doping method, the chemical dopants must match the energy levels for sample oxidation. For p-type doping, the electron affinity (EA) of the dopant should be higher than the ionization energy (IE) of the semiconducting polymer [26]. One typical way of calculating the IE of the conducting polymer is using electrochemistry and more specifically cyclic voltammetry (CV). P3HT films begin to oxidize at approximately 0 V versus the ferrocene/ferrocenium reference redox couple in TBAPF_6_ acetonitrile electrolytes [28]. This potential corresponds to the onset of p-type doping and can be referred to an ionization potential of IE ≈ 5.1 eV.

Chemical dopants such as Magic Blue (EA≈ 5.9 eV) [11] and 2,3,5,6-tetrafluor-7,7,8,8-tetracyanochin (EA ≈ 5.2 eV) [13, 16, 27] have for example appropriate electron affinities to dope P3HT. The absorption spectra of F4TCNQ-doped thin films indicate that the polymers predominantly form polaronic states, whereas F4TCNQ-doped P3HT solutions also give evidence for bipolaronic states [29]. Other chemical dopants for achieving highly doped states in P3HT include FeCl_3_ [30] or the organic salt dimesityl borinium tetrakis(penta-fluorophenyl)borate (Mes_2_B[B(C_6_F_5_)_4_]^−^) [31].

**Scheme 1:**
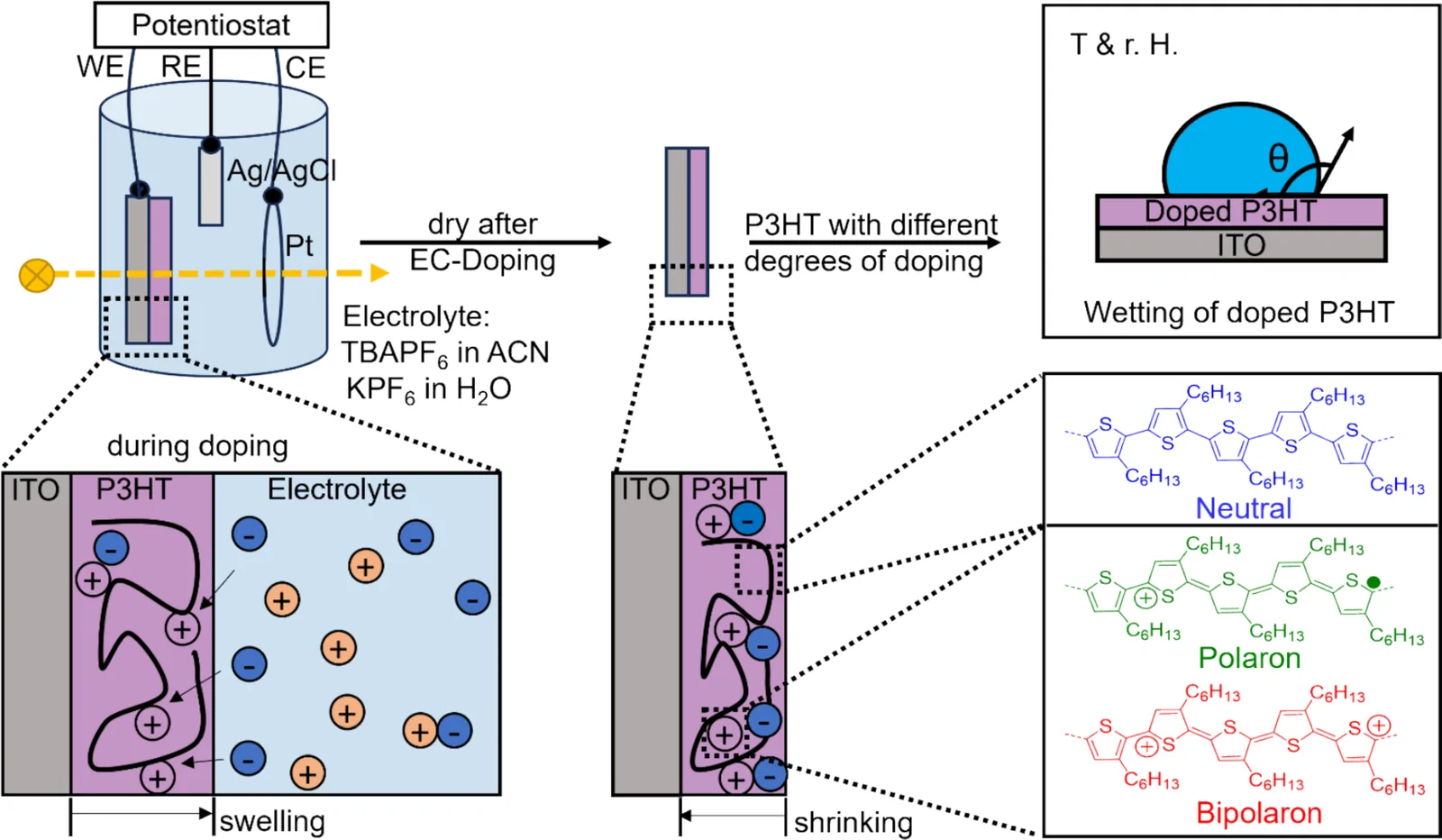
Schematic overview of an electrochemical doping process with a three-electrode electrochemical cell, consisting of a P3HT film on an ITO substrate as the working electrode (WE), a Ag/AgCl reference electrode (RE), and a Pt counter electrode (CE) immersed in electrolyte (TBAPF_6_ in acetonitrile /ACN or KPF_6_ in H_2_O). Thin film swelling takes place in the electrolyte during doping (EC-doping). After doping the films are removed and allowed to dry, which is accompanied by film shrinkage due to solvent evaporation. The doped films are then characterized with water contact angle measurements to determine the film wetting properties as a function of the doping potential

Given its doping efficiency and practicality, we chose electrochemical doping, as it allows us to tune the doping states by simply modulating the applied potential and accessing a wider range of oxidation states of the polymers [9, 10]. Previous studies from our group confirm that electrochemical doping is a powerful strategy to obtain doped films, transitioning from the neutral state via the polaron to the highly doped bipolaron state, which remain stable even after drying in the solid-state (Scheme 1). [9, 32]

In this study, we place electrochemically doped conjugated polymer films within the broader context of dynamic wetting on adaptive, flexible, and switchable substrates, where external stimuli are used to control interfacial properties. Electrochemical doping provides a direct route to tune both the electronic state and surface characteristics of conducting polymers, making them an excellent model system for responsive wetting interfaces. Within this frame, we systematically investigate the relationship between the doping potential and the resulting doped states of P3HT films by cyclic voltammetry coupled with in situ UV–Vis–NIR spectroscopy (Scheme 1). Building on this foundation, films in targeted doped states are prepared via potentiostatic doping in an electrolyte, followed by drying and subsequent characterization to assess their solid-state properties. Opto-electronic properties of the doped films confirm the oxidation states in solid-state. The entire process is conducted within a glovebox system to ensure an inert gas atmosphere. For wetting measurements, these doped films are transferred from the glovebox to a humidity- and temperature-controlled chamber where water contact angle measurements are performed to minimize atmospheric influences and ensure reproducible data. The top morphology of the doped films is characterized using atomic force microscopy (AFM) and scanning electron microscopy (SEM) and correlated with the water contact angle data. Complementary grazing-incidence wide-angle X-ray scattering (GIWAXS) is used to characterize the lattice variations within the crystalline domains under different doping levels.

## Results and discussion

### In situ spectroelectrochemical characterization of P3HT films

To understand the electrochemical doping process of P3HT thin films, cyclic voltammetry (CV) measurements were performed in a three-electrode configuration coupled with a UV–Vis–NIR spectrometer for real-time spectral recording. In situ spectroelectrochemistry allows the clear differentiation of doped species at specific applied potentials. The P3HT films were prepared by spincoating from 1,2-dichlorobenzene (DCB) solutions on top of the ITO working electrode (WE) and Pt works as counter electrode (CE). Here, two electrolyte systems were used to electrochemically oxidize thin P3HT films which are: TBAPF_6_ in acetonitrile (ACN) as organic electrolyte and KPF_6_ in water (H_2_O). Different oxidative regimes were accessed in these two systems, corresponding to their specific electrochemical windows. For acetonitrile, the measurements were conducted with a Ag/AgCl wire as reference electrode (RE) and afterward referenced versus Fc/Fc⁺. Potentials between − 0.8 V and 0.8 V versus Fc/Fc⁺ were chosen for the ACN electrolyte, while in the aqueous electrolyte, the potential range was between − 0.2 and 1.4 V using a commercially available Ag/AgCl reference electrode. The application of higher doping potentials was avoided to prevent irreversible side reactions [33].

**Fig. 1:**
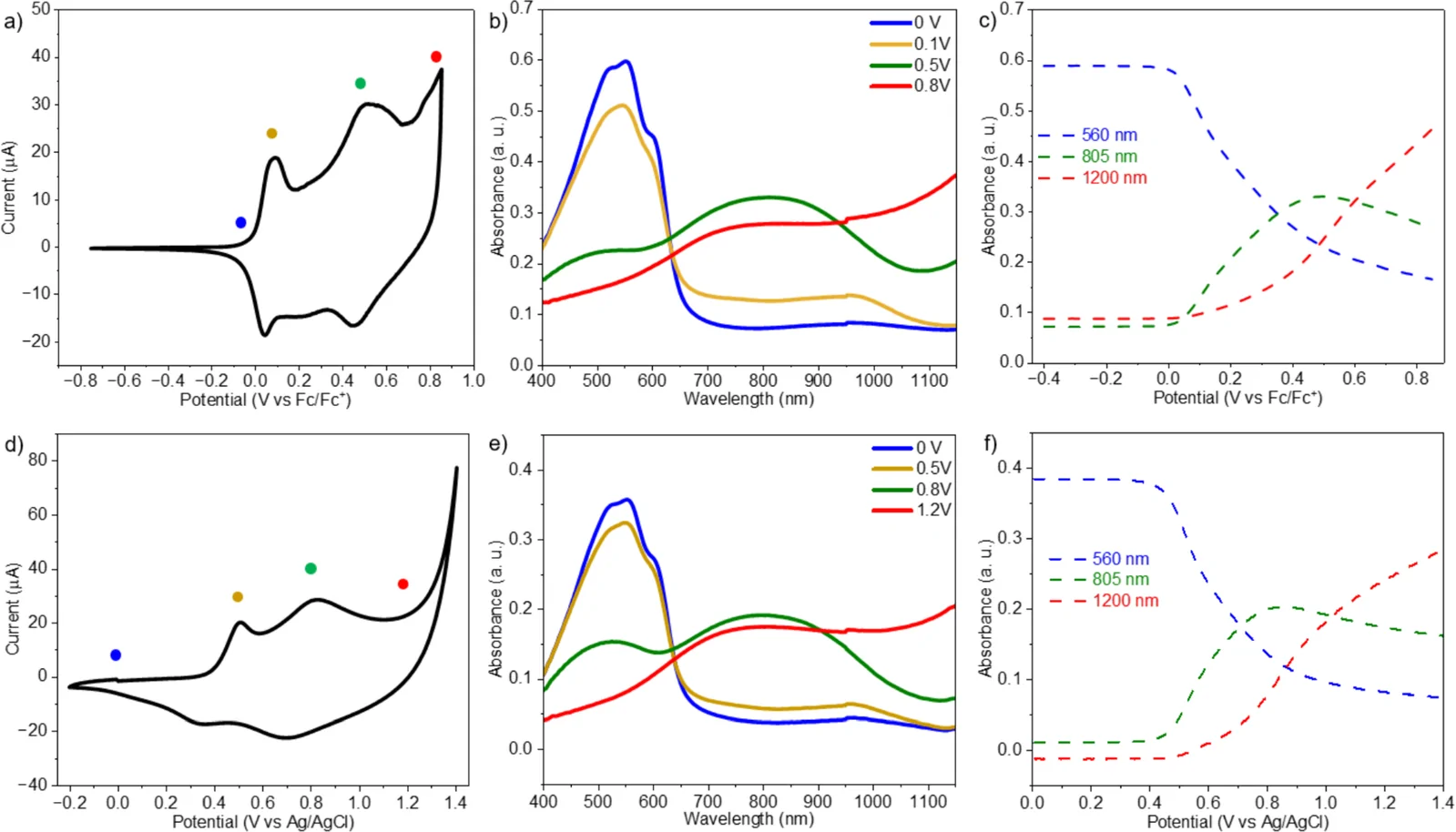
In situ spectroelectrochemical studies of P3HT films prepared from DCB solution. **a** CV measured in 0.1 M TBAPF_6_/ACN at 20 mV/s on an ITO electrode; 2nd cycle is shown. **b** UV–vis–NIR spectra obtained at the color-highlighted potentials in (**a**), and **c** spectral evolution of the absorption maxima at 560, 805, and 1200 nm corresponding to the characteristic species in the forward scan from (**a**). **d** CV measured in 0.1 M KPF_6_/H_2_O at 20 mV/s on an ITO electrode; 2nd cycle is shown; **e** with correlating UV–vis–NIR spectra at the color-highlighted potentials in (**d**), and **f** spectral evolution of the absorption maxima at 560, 805, and 1200 nm corresponding to the characteristic species in the forward scan from (**d**)

The upper panels of Fig. 1 present the recorded cyclic voltammograms (Fig. 1a) and the respective UV–Vis–NIR spectral analysis (Fig. 1b, c) from the in situ spectroelectrochemical studies performed in TBAPF_6_/ACN. The colored absorption spectra in Fig. 1b correspond to specific potentials highlighted by colored points in Fig. 1a in the forward scan of the second cycle. The evaluation of the intensity of the absorption maxima (Fig. 1c) at characteristic wavelengths clearly shows the presence and changes of three distinct species: a neutral state peak at 560 nm, a polaron state peak at 805 nm, and a broad bipolaron state absorption at wavelengths of 1200 nm and higher [9, 10, 12].

The blue point in Fig. 1a corresponds to the blue absorption curve in Fig. 1b, which exhibits a significant characteristic peak around 560 nm in the neutral state. Here, a *λ*_max_ value around 520 nm is typically attributed to the amorphous phase, while the maxima centered at 560 nm and 605 nm (corresponding to the 0–0 and 0–1 vibronic bands) are primarily attributed to the crystalline phase [10, 34].

For the yellow point at 0.2 V in Fig. 1a, the corresponding yellow curve of the UV–Vis spectrum reveals an overall decrease in the neutral state peaks (520 nm, 560 nm, 605 nm) and a concomitant increase in the polaron state peak at 805 nm, indicating the conversion from neutral to polaron states [8, 34]. The more pronounced shoulder peak elimination at 605 nm during oxidation aligns with the findings of Cavassin et al., which attributed this phenomenon to the preferential location of polaron states within the crystalline regions of the polymer [10]. Further doping at 0.5 V (green point) and 0.8 V (red point), along with the corresponding curves, reveals that the polaron species first increases and then decreases, while bipolaron species continue to increase. It is important to note that at higher doping potentials (red curve) the polaron state is still present, marked by a broad shoulder around 800 nm, suggesting the coexistence of these two oxidation states. The attribution of absorption bands in the UV–Vis–NIR spectra has been a matter of debate in the literature. Recent studies have employed DFT calculations to address this. While Sahalianov et al. attribute the two observable bands around 800 and 1600 nm to both oxidation states (polaron and bipolaron) [35], a TD-DFT/NTO analysis by Wu et al. supports the traditional attribution of the bands at 800 and 1600 nm to the polaron and bipolaron states, respectively [36]. From our point-of-view one can clearly state that the neutral P3HT films can be oxidized into two oxidation states from the absorbance spectra in Fig. 1b.

Initial attempts with KCl in water as electrolyte yielded no typical oxidation behavior, likely due to the tightly bound hydration shell around Cl^−^ hindering its penetration into the rather hydrophobic P3HT film [37, 38]. Consequently, in situ spectroelectrochemical measurements were performed for P3HT doped in a KPF_6_/H_2_O electrolyte (Fig. 1d–f), showing similar behavior to that obtained in the TBAPF_6_/ACN electrolyte. The first (yellow point) and second (green point) oxidation peaks in the voltammogram (Fig. 1d), located at 0.4 V and 0.8 V (vs. Ag/AgCl), respectively, can be attributed to increased oxidation to polaronic states. The third peak (red point) is again attributed to the formation of bipolaron species. The obtained real-time absorption spectra (Fig. 1e, f) confirm this hypothesis, showing consistent behavior with previous studies [10, 38, 39].

Regarding the peak separation of the polaron peak in the cyclic voltammograms (found both in Fig. 1a, d) Skompska et al. reported a similar behavior in their investigation of the scan-rate dependence of P3HT and poly(3-dodecylthiophene) with varying regioregularity, where this behavior was ascribed to differences in conjugation length [40]. This interpretation is consistent with earlier findings for poly(3-methylthiophene), in which the first oxidation wave was assigned to ordered domains with longer conjugation lengths, while the second oxidation wave was associated with disordered domains characterized by shorter conjugation lengths [41]. Overlapping multiple oxidation peaks have been found to be more dominant also for polymers with increasing regioregularity [42]. Furthermore, Bruchlos et al. reported an even more distinct peak separation in highly ordered morphologies obtained via crystallization methods, attributing this effect to the presence of well-defined ordered domains with extended conjugation [28].

Several peaks can be resolved during the backward scan of the CV curves in Fig. 1a, d, corresponding to the reduction of bipolaron and polaron states back to the neutral state. The phenomenon in which charge injection exceeds charge release was also reported by Neusser et al. [9] and Hornberger et al. [32] when investigating electrochemical doping of disordered P3HT films cast from chloroform (CF) solutions and attributed to charge trapping effects. Further comparison of multiple CV cycles indicates that P3HT doped in the KPF_6_/H_2_O system exhibits less pronounced first cycle effects and higher reversibility across the first three cycles (Figure S1), suggesting less morphological disruption during doping and dedoping compared to doping in the TBAPF_6_/acetonitrile system. One explanation could be that the hydrophobic side chain of P3HT might hinder water molecule injection, resulting in minimal microstructural changes and reduced expansion of the P3HT lattice [37, 45]. This could further affect the incorporation of the ions into the polymer matrix. Note that the generation of doped species in electrochemical experiments is always accompanied by ion charge compensation. It is fair to assume that PF_6_-anions get incorporated both in the case of the organic and the aqueous electrolyte [46].

### Opto-electronic properties of electrochemically doped P3HT films in the solid state

Leveraging the information from in situ spectroelectrochemical data, P3HT films with desired doping levels can be targeted through the application of a controlled potential, a method also known as potentiostatic doping. Subsequently, the opto-electronic properties of these potentiostatically doped films, once prepared by electrochemical doping and dried, can be further investigated in the solid state. However, direct conductivity measurements for P3HT films on ITO substrates are not feasible. Thus, a glass substrate coated with four gold lines was used [9, 47]. A detailed illustration of the measurement can be found in Figure S2. Absorption measurements were performed directly on the glass region of the modified substrate, whereas conductivity was carried out on the gold regions using a four-point probe configuration.

The chronoamperometric curves recorded during potentiostatic doping reveal that higher applied potentials result in higher initial currents (Figure S3a). This observation indicates that a larger amount of polymer material undergoes doping, resulting in a higher number of oxidized states. This is accompanied by an increased ion migration and incorporation into the films to achieve charge neutrality. Notably, all chronoamperometric curves decay to approximately zero within 5 s, indicating the completion of the doping process. Figure S3b shows the change in hole density as a function of doping potential, determined by integrating the chronoamperometric curves. Generally, the hole density in films doped below 0 V is approximately one order of magnitude lower than that in films doped between 0 and 0.4 V. At potentials above 0.4 V, the hole density reaches a plateau approximately one order of magnitude higher. The data is consistent with findings in the literature regarding CF-prepared P3HT films [9], suggesting similar changes in electronic properties within this range for DCB-prepared films.

**Fig. 2:**
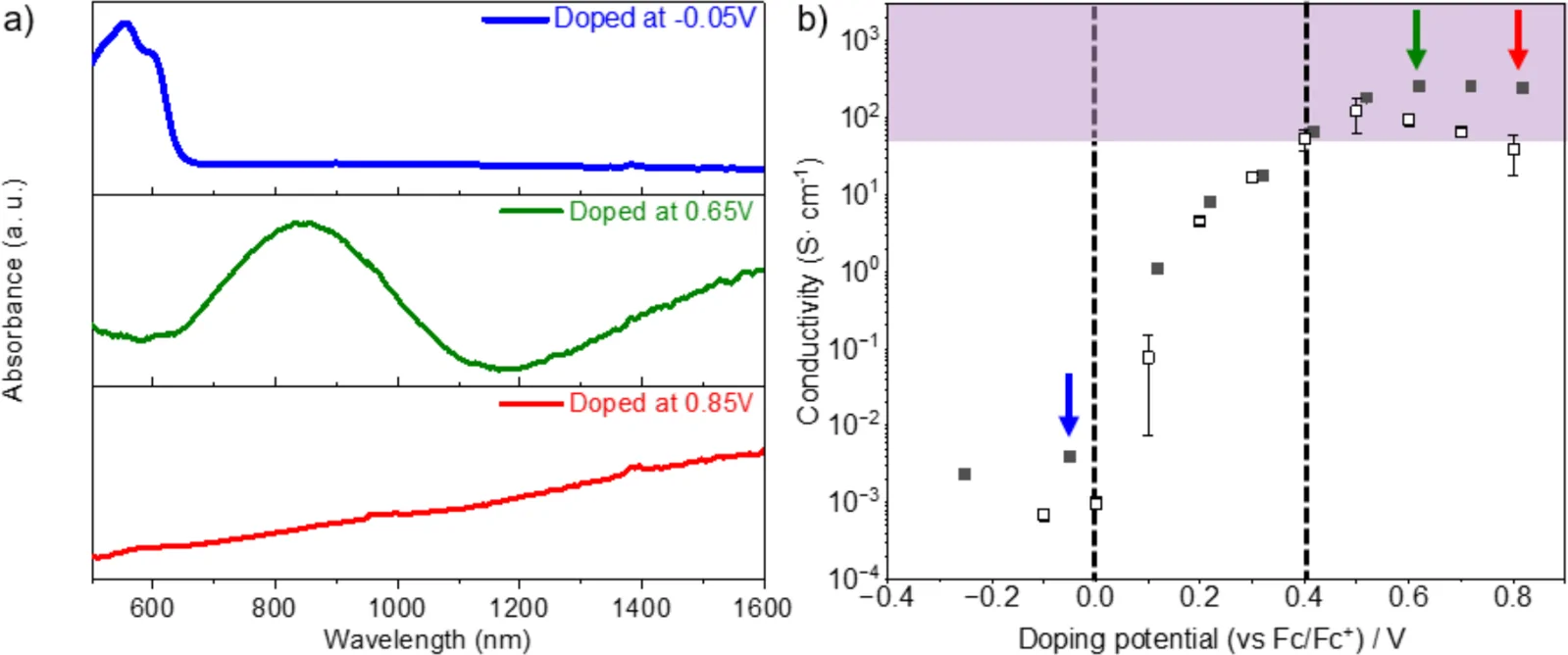
Optical and electronic properties of electrochemically doped P3HT film**s** (**a**) Ex situ solid-state absorption measurements performed after doping at three different doping potentials for films spincoated from DCB solutions onto Au-coated glass electrodes. **b** Conductivity of electrochemically doped P3HT films prepared from DCB solution (■) and p-xylene solution (□) on 4-line Au electrodes

Figure 2a shows that the absorption spectrum of the film doped at − 0.05 V (blue curve) exhibits a clear peak at 560 nm which is indicative of the neutral state. Conversely, the green curve indicates that P3HT doped at 0.4 V is predominantly in the polaron state, while the red curve reveals that P3HT doped at 0.8 V is predominantly in the bipolaron state. These ex situ absorbance spectra from the solid-state film are consistent with the in situ spectroelectrochemistry of the film doped in TBAPF_6_/ACN, suggesting a successful strategy for obtaining stable doped states. Regarding conductivity of the DCB-prepared film, Fig. 2b, films doped below 0 V exhibited low values, which are indicative of a neutral state of P3HT. Conductivity increased significantly in the doping range of 0–0.4 V, subsequently reaching a plateau. For DCB-prepared P3HT doped films, a maximum achieved conductivity of 258 ± 2 S/cm was obtained at 0.65 V. This observed conductivity value exceeds those previously reported for disordered P3HT films spincoated from chloroform solutions [9]. This enhancement is likely attributable to the increased crystallinity, influenced by the high-boiling-point solvent employed [6]. It is important to note that both the crystalline and the amorphous areas are beneficial for high conductivity [9, 10]. According to Cavassin et al., the former provides conductivity pathways and fast doping kinetics, while the latter offers higher charge density through the formation of bipolarons and allowing for significant ionic uptake [10].

As a comparison, we report the electrical conductivity of doped P3HT thin films prepared from p-xylene (Fig. 2b) which is known to yield highly crystalline films [48]. Conductivity is of the same order of magnitude and exhibits similar trends with increasing doping potential. This supports our conclusion, namely that crystallinity is not the sole factor influencing charge transport, rather, a well-connected network of percolation paths between these regions appears essential [49, 50].

### Wetting behavior of electrochemically doped P3HT films prepared in organic electrolytes

**Fig. 3:**
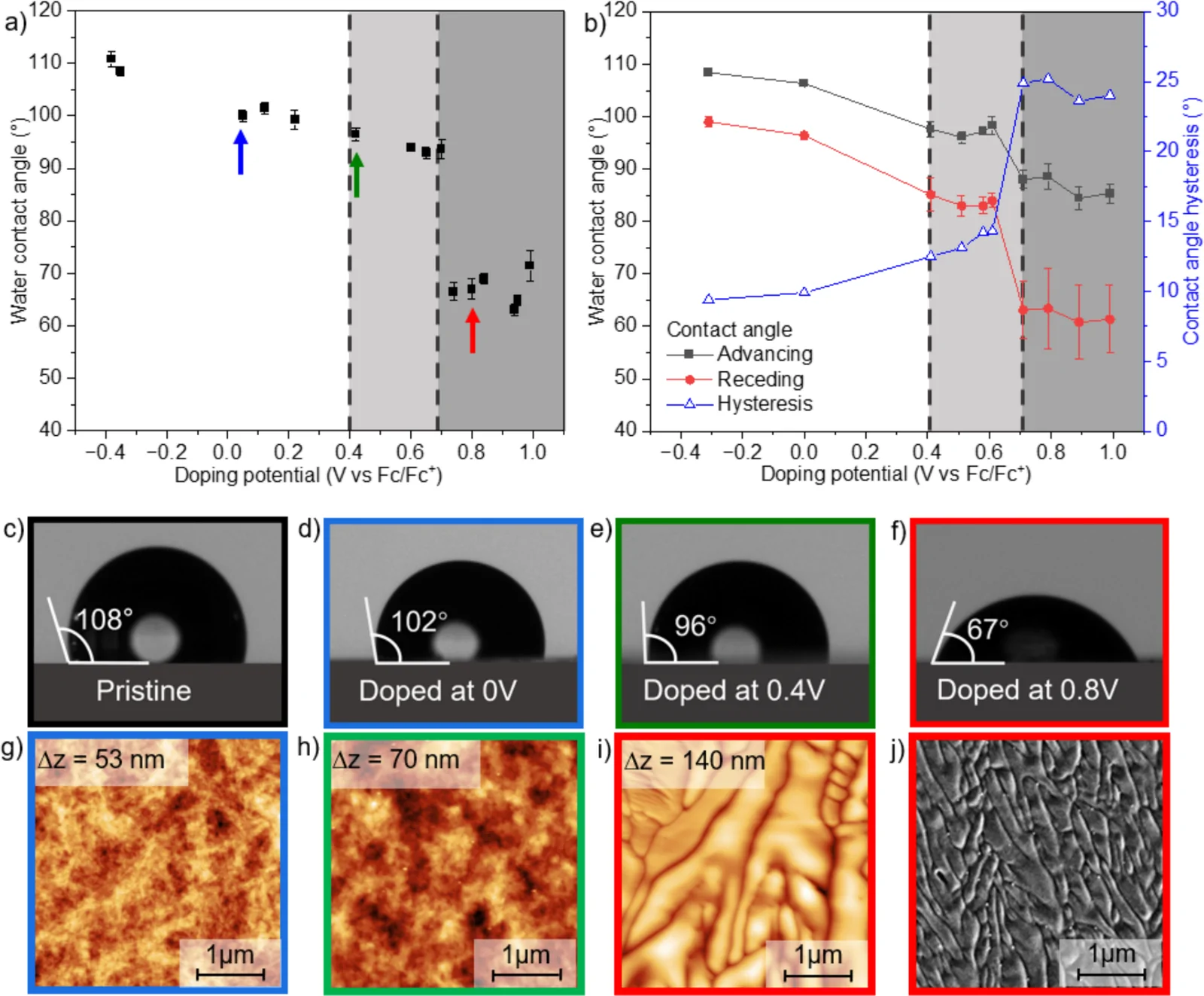
Wetting properties and morphology of electrochemically doped P3HT films prepared from DCB solution (electrochemical doping in TBAPF_6_/acetonitrile, according to Fig. 1). **a** Static and **b** dynamic water contact angle measurements of electrochemically doped dry films on ITO substrates. **c**–**f** Water contact angle images of pristine and doped films at 0 V, 0.4 V, and 0.8 V vs. Fc/Fc⁺. **g**–**i** AFM images of films doped at 0 V, 0.4 V, and 0.8 V. **j** SEM image of a film doped at 0.8 V vs. Fc/Fc⁺

To investigate the impact of electrochemical doping on surface properties, P3HT films deposited on ITO were potentiostatically doped at various potentials for 40 s and dried overnight in an inert atmosphere. Afterward, the water contact angle (WCA) of the films was measured to quantify the changes in surface energy. The absorption spectra of these doped solid-state films (Figure S4) were measured before the WCA measurements to confirm the targeted oxidation states. The contact angle measurements were performed inside a custom-built setup, ensuring controlled relative humidity and temperature conditions. The obtained static contact angles as a function of the applied doping potential are summarized in Fig. 3a. Figure 3d–f shows the corresponding images of the water droplets for the pristine and doped films (shown for doping potentials of 0, 0.4 and 0.8 V). The pristine film, included in Fig. 3c, shows a WCA of 108°, classifying pristine P3HT as having a hydrophobic surface.

For films doped below 0.4 V (vs. Fc/Fc⁺), the water contact angle decreased from 110° to 96°. The corresponding absorbance spectra recorded for this range revealed the formation of the polaron state from the neutral state. Further increasing the doping potential from 0.4 to 0.7 V resulted in a further decrease to 92°, correlating with the formation of the bipolaron state, though polaron states are still present. Notably, for the films doped at potentials above 0.7 V, i.e., in the presence of mainly bipolaronic species, a pronounced reduction in contact angle was observed, with values as low as 67°. This implies that the P3HT surface can be switched from a more hydrophobic surface to a hydrophilic surface by application of doping potentials higher than 0.7 V. This can be partially explained by the polarization of the polythiophene backbone due to oxidation. In the literature, changes in surface energy and water contact angle are related to the polarization of the conducting polymers, with a strong participation of the counterions. For the special case of dodecylbenzenesulfonic acid (DBSA)–doped polyaniline (PANI), oxidation was shown to result in a more hydrophobic surface. This was explained by an increase in the positive charge density along the backbone upon oxidation of PANI, with DBSA molecules orienting their polar negative sulfonate groups toward the polymer backbone and exposing their hydrophobic alkyl chains at the surface [23, 24]. Upon reduction in DBSA:PANI, loosely bound DBSA was proposed to induce a more polar surface due to their interaction with the water droplet on top. Correspondingly, the formation of charged P3HT chains (polarons/bipolarons) and their associated counterions (in our case PF_6_^−^) might render the thin film more hydrophilic [17, 23]. A further study shows that the presence and length of side chains in polythiophene compounds also influence this behavior. In particular, increasing the alkyl side-chain length can lead to larger variations in the water contact angle under similar applied doping potentials [24]. However, this explanation does not account for the significant decrease observed in highly doped P3HT films (above 0.7 V vs. Fc/Fc⁺).

The potential-dependent wetting behavior is also visible in advancing and receding contact angle measurements, as depicted in Fig. 3b. This figure illustrates the evolution of the advancing and receding contact angle and the hysteresis, defined as the difference between the advancing and receding contact angles [51]. With increasing doping potential, the advancing and receding contact angles were observed to decrease in the same potential range, with the receding contact angle consistently presenting lower values. A similar significant reduction takes place at doping potentials at 0.7 V, after which the contact angle is approximately constant. This reduction is particularly pronounced for the receding contact angle, resulting in a large contact angle hysteresis.

**Fig. 4:**
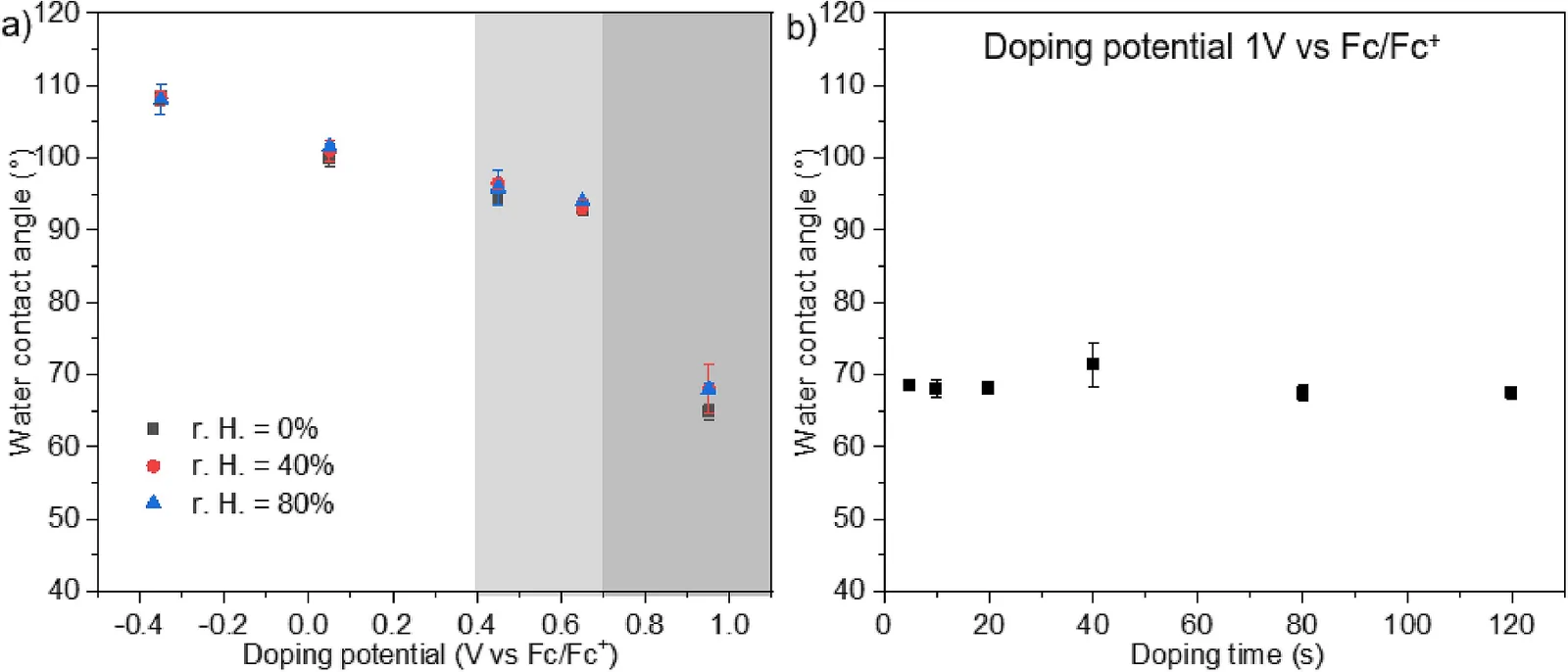
Factors influencing the wetting properties of electrochemically doped P3HT films prepared from DCB solution. **a** Water contact angle measurements at different r.H. conditions; **b** Influence of the doping time for doping at 1 V

One critical consideration is whether the observed reduction in contact angle originates from intrinsic surface wettability or from water adsorption into the bulk of the doped films. To demonstrate that the doped P3HT surface remains passive and non-hygroscopic, we measured water contact angles across a broad range of relative humidity (r.H.). The maximum r.H. was around 80%, measurements at (super)saturated atmospheres were not topic of this study. Prior to the measurement, films were stored in the chamber at the respective r.H. for 30 min to be in equilibrium. As shown in Fig. 4a, the static water contact angle values remained constant as the relative humidity (r.H.) increased from 0 to 80%. Similarly, the dynamic contact angle remained constant (Figure S5) within the chosen r.H. regime. This stability across varying moisture levels suggests that the film doped in the chosen range does not undergo significant water uptake, which means that the measured contact angles reflect surface energy rather than bulk water adsorption of the hydrophobic surface of P3HT.

Since the surfaces are, however, not passive but can interact with overlying water, P3HT emerges as a promising material for tunable wetting, externally controllable by an applied potential. The next question addresses the duration time required for the transition from a hydrophobic surface (108°) to a hydrophilic surface (67°) at high doping potentials. Here, we investigated the water contact angle on films by varying the doping duration time at the same potential, specifically at 1 V vs. Fc/Fc⁺ which is in the range where polarons and bipolarons are present. From Fig. 4b, a doping duration of 5 s is deemed sufficient to achieve the desired hydrophilicity changes. This observation is corroborated by the chronoamperometric curves, which indicate that the majority of oxidation and accompanying ion injection does occur within the initial 5 s. This suggests that P3HT serves as a promising material for applications requiring fast changes in the wetting properties from hydrophobic to hydrophilic.

### Morphology of electrochemically doped P3HT films prepared in organic electrolytes

The static and dynamic WCA measurements, along with the observed hysteresis, suggest significant chemical and/or morphological changes at the thin film surface, especially at high doping potentials. To understand these phenomena, morphological studies of the doped films were performed. Figure 3g–i shows topographic AFM images of thin films doped at different oxidation states: neutral, polaron (doped at 0.4 V vs Fc/Fc^+^), and with predominantly bipolaron species (doped at 0.8 V vs Fc/Fc^+^). For the neutral P3HT film, the surface morphology exhibits low order, whereas the presence of crystalline domains is confirmed through the absorption spectra (Figure S4), which exhibited characteristic peaks at 560 nm and 605 nm. Thin film height variation (Δz) and root-mean-square roughness (Rq) were extracted from the AFM scans to quantitatively evaluate morphological changes. Neutral films (Fig. 3g) and films doped up to the polaronic state (Fig. 3h) exhibited similar Rq values of approximately 2 nm, and Δ*z* values ranging from 55 to 70 nm. In general, for films doped below 0.7 V (vs Fc/Fc^+^), the similar smooth and flat surfaces suggest that the change in the static water contact angle (from 110° to 92°) at this range is not significantly influenced by surface topography. The minor effect on morphological changes during the transition from the neutral to polaronic states has also been reported for P3HT films chemically doped by F4TCNQ [52, 53]. At such doping states, the polymer chains have very limited freedom to rearrange.

The most significant morphological changes occur when the thin films reach high doping levels (over 0.7 V vs Fc/Fc^+^), these changes coincide with water contact angle evolution and the sharp drop in WCA to values below 70°. These morphological features are seen in Fig. 3i where various creases are present across the entire doped film surface. Some displaying a valley-like morphology with large height differences of up to 100 nm, while others show smaller creases with variations around 20 nm. The significant topographic variations and correspondingly higher surface roughness of approximately 11 nm could be responsible for the sharp changes observed in the static and dynamic contact angle measurement. This morphology was further confirmed by SEM imaging, as shown in Fig. 3j.

To explain this, the formation of creases on the surface of solid-state doped films can be separated into two stages. The first stage occurs during electrochemical doping, where ions need to be incorporated to ensure electroneutrality, often accompanied by solvent molecules entering the film and causing the film to swell in the electrolyte solution. Skompska et al. reported an increase in film thickness by up to 17% in the thickness of thick P3HT films (3–3.5 µm) relative to the pristine state, an effect attributed to the expansion of the polymer structure via the uncoiling of twisted polymer chains during doping in 0.1 M LiClO_4_ in propylene carbonate (PC), as measured by in situ AFM [54]. Their study also showed that swelling is insignificant at low doping potentials, then rapidly increases after a certain point, followed by a plateau. This swelling effect is even more pronounced in thin films, as Chao et al. observed an increase of 10 nm in poly(3-methylthiophene) films with an initial thickness of 19 nm [55]. The second stage is when the films were taken out of the electrolyte solution, drying rapidly due to the swift evaporation of the solvent. Surface creases are generally associated with mechanical instabilities that develop when the film undergoes substantial swelling, e.g., observed in gels. Upon drying, the swollen film contracts; however, the crease morphology can be retained due to residual stresses, irreversible structural rearrangements, or surface pinning effects generated during the swelling–deswelling cycle [56–58]. The observed wetting behavior of the doped P3HT films suggests an applicability of the Wenzel model, in which the liquid follows the surface topography of the roughened film [59]. The presence of water in the surface creases can also explain the large contact angle hysteresis in the dynamic measurements, leading to a pinning effect of the droplet edge [60]. This results in a smaller receding contact angle compared to the advancing contact angle, producing large hysteresis. This effect is also reflected in the standard deviation of these measurements, which was significantly higher for the receding contact angle measurements at high doping potentials (Fig. 3b, over 0.7 V vs Fc/Fc^+^).

In addition to the creases and valley-like features, the AFM and SEM images also revealed the presence of small particle deposits located primarily around areas where valley bifurcations take place. These deposits could be composed by excess electrolyte salts on the surface stemming from drying after the electrochemical doping. Their preferential positioning along the creases could suggest that salt crystal nucleation can be part of the driving force for crease formation due to anisotropic film swelling. A similar nucleation effect has been previously reported in regiorandom P3HT films after multiple CV cycles in water-based electrolytes [61]. The electrolyte salts were found to aggregate on the boundary between doped and undoped regions, forming tall wall-like aggregates.

**Fig. 5:**
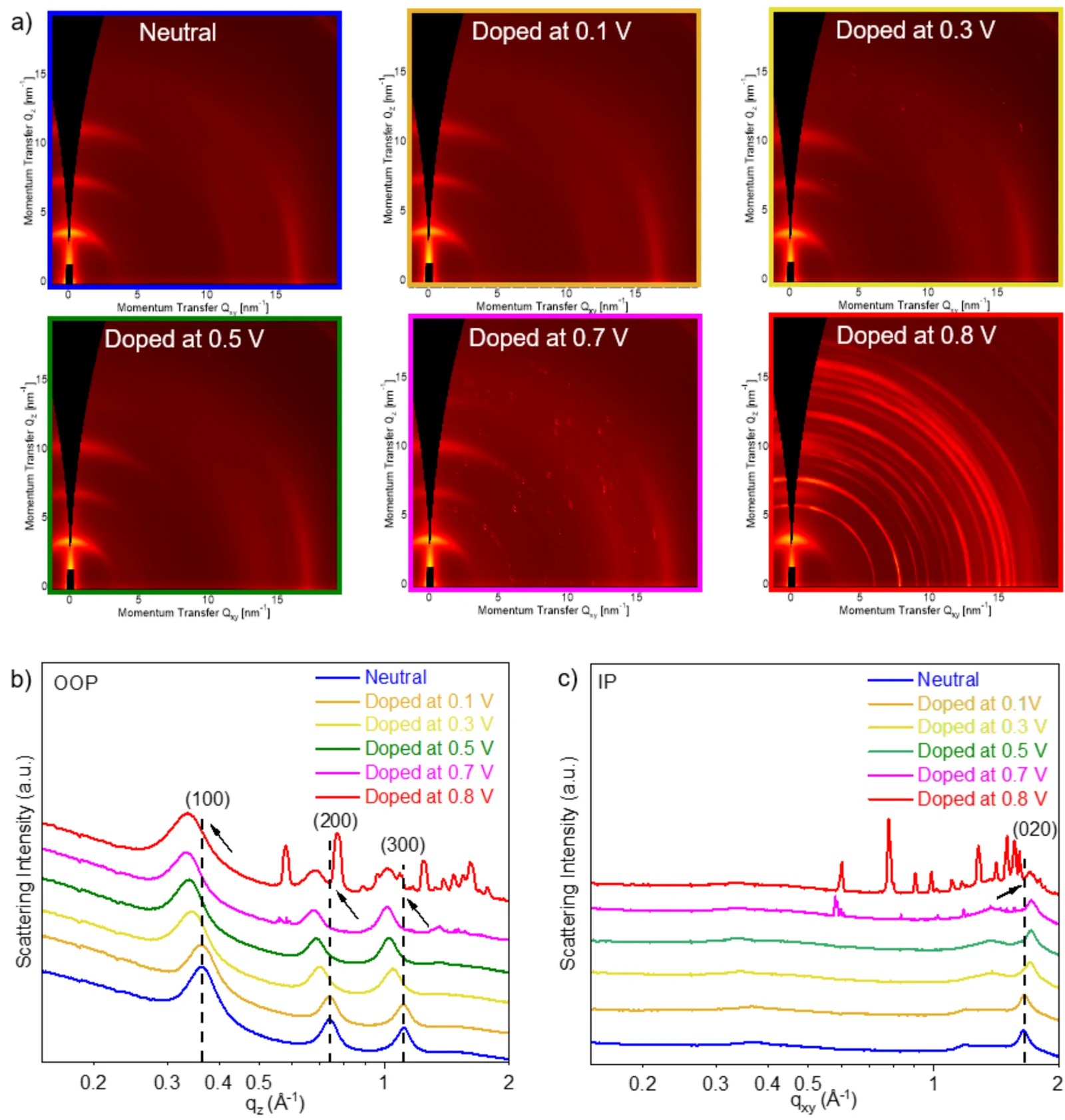
GIWAXS of electrochemically doped P3HT films prepared from DCB solution and doped in 0.1 M TBAPF_6_/ACN for 40 s at the given potentials. **a** 2D GIWAXS images of films doped at different potentials vs. Fc/Fc⁺. **b** 1D out-of-plane (OOP) and **c** in-plane (IP) diffraction profiles

To further understand the microstructure changes in electrochemically doped P3HT films, additional morphological characterization was carried out using GIWAXS. This technique is well-suited for characterizing semi-crystalline P3HT which comprises crystalline and amorphous regions [5]. Neutral P3HT films prepared from a DCB solution primarily exhibit an edge-on orientation. This is strongly supported by GIWAXS data, in which prominent h00 out-of-plane lamellar peaks (e.g., (100), (200), (300)) and an in-plane *π*–*π* peak ((020)) collectively confirm a well-defined edge-on structure (Fig. 5a–c). For DCB-prepared films, lamellar stacking exhibits a spacing of approximately 1.75 nm, corresponding to a (100) peak at *q* ≈ 0.36 Å^−^ [1]. Similarly, aromatic (*π*–*π*) stacking exhibits a spacing of approximately 0.38 nm, resulting in a (020) peak at *q* ≈ 1.65 Å^−^ [1]. These values fall within the typical range for P3HT reported in the literature [6, 62].

We further investigated the changes in the structural characteristics as function of electrochemical doping potential (Fig. 5). Analysis of the out-of-plane (OOP) diffraction patterns revealed that an increase in doping potential resulted in a shift of the (h00) peak to lower *q*, indicating an expansion in lamellar stacking distance from 1.75 to 1.9 nm as function of potential. Conversely, analysis of the in-plane (IP) diffraction patterns exhibited a shift of the (020) peak to higher *q*, signifying a decrease in the *π*–*π* stacking distance from 0.38 to 0.36 nm. This shortening of the aromatic (*π*–*π*) stacking is consistent with the observed significant increase in conductivity via electrochemical doping. Additional diffraction peaks emerged in films at high doping levels, and these peaks are attributed to the incorporated electrolyte salt species. Their intensity increased progressively with increasing doping potentials. Importantly, the edge-on orientation peak remained largely preserved, suggesting that the overall crystalline texture was maintained after electrochemically doping even at high doping levels.

The observed increase in lamellar stacking distance due to ion insertion aligns with a previous study, which reported that chemical doping can increase the lamellar distance (e.g., from 1.6 to 1.8 nm) [63]. The most significant change was observed for the film doped between 0.1 and 0.3 V vs. Fc/Fc⁺. Minor changes followed until 0.7 V, after which the behavior stabilized, primarily corresponding to the formation of the polaron state as evidenced by the in situ spectroelectrochemistry results shown in Fig. 1. The formation of bipolaron states did not induce further significant changes in the crystalline part. This observation aligns with previous in situ GIWAXS observations when inducing bipolarons to P3HT by doping with solid electrolytes based on [EMIM][TFSI] [64]. This finding also supports the statement that bipolarons preferentially form in amorphous regions, while polarons are preferred in the crystalline region [10].

### Wetting and morphology of electrochemically doped P3HT films prepared in aqueous electrolytes

**Fig. 6:**
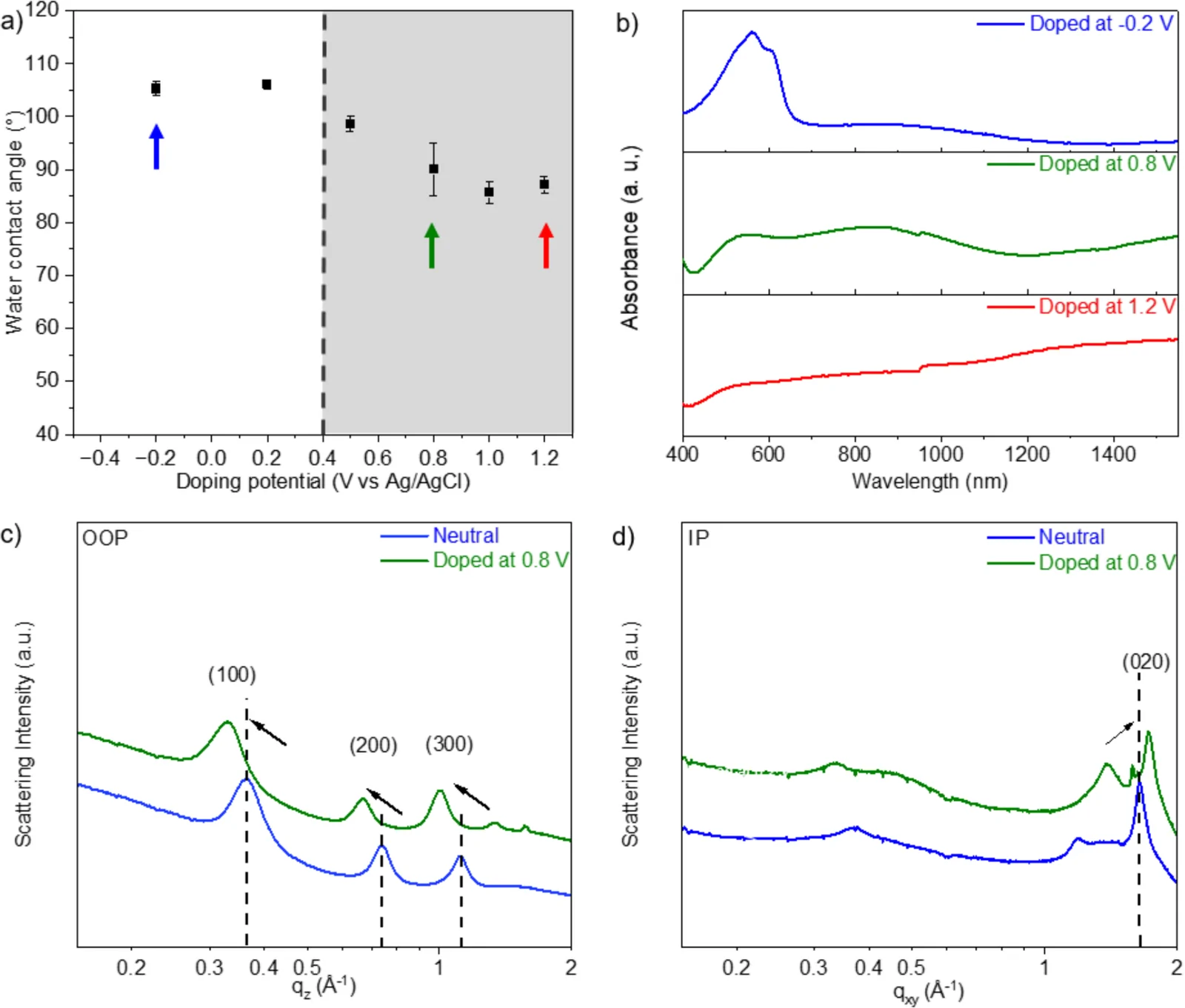
Wetting properties and morphology of electrochemically doped P3HT films prepared from DCB solution (electrochemical doping in KPF_6_/H_2_O, according to Fig. 1). **a** Static and water contact angle measurements of electrochemically doped dry films on ITO substrates **b** Ex situ solid-state absorption measurements performed after doping at three different doping potentials vs. a commercial Ag/AgCl reference electrode **c** 1D GIWAXS out-of-plane (OOP) and **d** in-plane (IP) diffraction

The prevalence of water in biomedical applications, especially biosensors, motivated us to further investigate aqueous electrolyte systems. Therefore, we examined the wettability and morphology of electrochemically doped P3HT films after utilizing aqueous electrolytes for electrochemical doping. This approach contrasts with the previously discussed surface behavior after using an acetonitrile electrolyte. In this case, the hydrophobic polymer film surface can achieve the same doping states (Fig. 1) but seems to mitigate film swelling during the doping process. It is important to note that standard aqueous electrolytes, such as KCl, fail to facilitate stable doping for P3HT films. This can likely be attributed to the strongly bound hydration shell surrounding Cl^−^, which impedes its penetration into the relatively hydrophobic P3HT film, a characteristic supported by the pristine film water contact angle of 108°. Alternatively, KPF_6_ proved effective. The wettability of films doped in KPF_6_/H_2_O (Fig. 6a) exhibited less pronounced changes compared to films doped in TBAPF_6_/ACN. The water contact angle remained constant at 106° below 0.4 V, corresponding to the neutral state indicated by the in situ and ex situ absorption spectra (Fig. 1f, Fig. 6b). It then decreased to 90° between 0.4 and 0.8 V, consistent with the formation of the polaron state, and subsequently stabilized at 87° upon going to higher potentials. The change in contact angle from 106° to 87° can be attributed to the same explanation proposed above for P3HT films doped from neutral state to the polaronic regime in an acetonitrile electrolyte, where the contact angle decreases from 108° to 92°. GIWAXS data (Fig. 6c, d) support this interpretation: for the film at 0.8 V in the KPF_6_/H_2_O aqueous system, a similar leftward shift of the (100) reflection and a rightward shift of the (020) reflection was observed. This behavior is consistent with our previous findings from the absorption spectra of the solid-state doped films, which show that P3HT can be driven into a polaronic state when doped at 0.8 V in the aqueous KPF_6_/H_2_O system (Fig. 6b). However, even at high doping levels, no significant changes in contact angle were observed. Complementary morphology studies support these observations. For P3HT films doped at 1.2 V versus a commercial Ag/AgCl reference electrode in KPF_6_/H_2_O electrolyte, which exhibited absorbance comparable to P3HT doped at 0.8 V versus Fc/Fc⁺ in TBAPF_6_/ACN (Figures S4), no wrinkle-like morphology was observed (Figure S7). This finding supports our assumption that sufficient swelling induced by ion uptake during the doping process is a necessary condition for the formation of the characteristic wrinkle-like structures. In the aqueous electrolyte system, the hydrophobic polymer film can still reach the same doping states, but the reduced swelling seems to suppress the development of these wrinkles. This explains why the creases are only observed in films doped at high potentials within the TBAPF_6_/ACN doping system. Similar conclusions were reported for thick P3HT films doped in a 0.1 M LiClO_4_ aqueous system which revealed significantly less swelling compared to a propylene carbonate system [54].

Another critical issue is the influence of the slow evaporation rate of residue water on the doped film. To evaluate this, the doped films were kept under N_2_ atmosphere and their stability was monitored by UV–Vis spectroscopy for 30 min. A gradual decrease in the absorption intensities associated with polaron and bipolaron species was observed over time, indicating a dedoping process in the films (Figure S8). This phenomenon was even more pronounced for the film doped at 1.2 V, for which the 1200 nm peak was no longer observed after 30 min. This indicates that bipolarons generated in aqueous solution appear to be unstable in solid state.

## Conclusion

We have systematically investigated the full range of accessible doping states of P3HT and correlated them with their wetting properties. By varying the applied doping potential across the entire window, a strong relationship between doping level, opto-electronic properties, and tunable wetting behavior in solid-state doped films was revealed. Beyond phenomenological observations, this work further clarifies the role of doping-induced microstructural evolution and its impact on material performance, as supported by the AFM, SEM, and GIWAXS analyses.

**Fig. 7:**
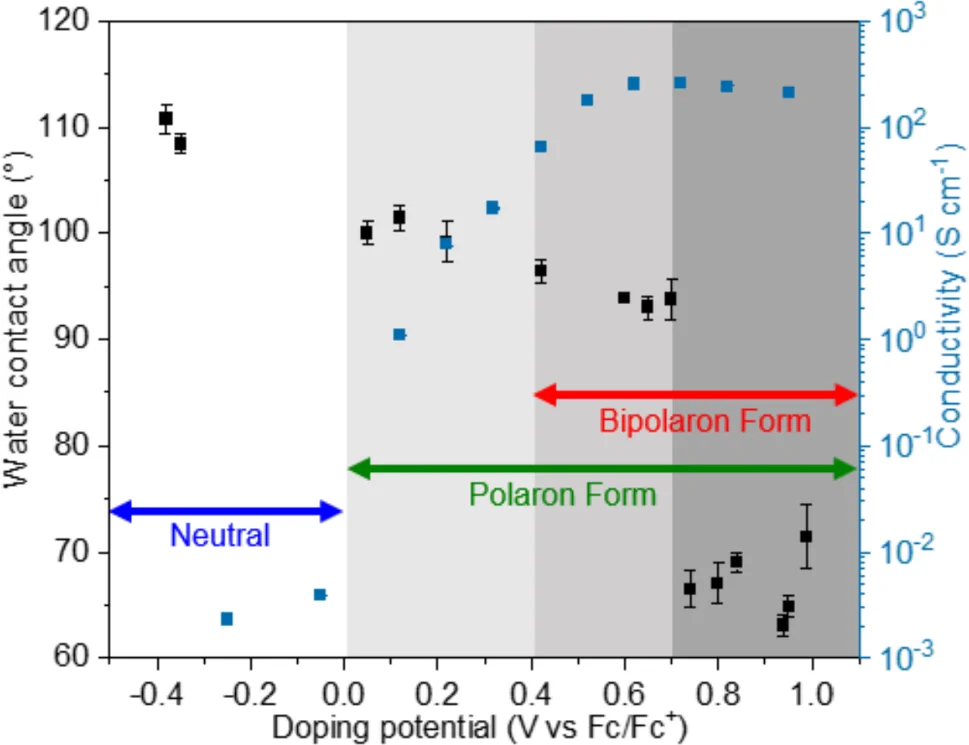
Summary of static water contact angle (■) and conductivity (■) of electrochemically doped P3HT films, which were prepared by spin-coating from DCB solutions on ITO substrates and subsequently doped in a TBAPF_6_ acetonitrile system

Compared to doping in KPF_6_/H_2_O aqueous systems, solid films prepared using the TBAPF_6_ in acetonitrile system exhibit higher stability and greater tunability in wetting behavior. This suggests enhanced potential for applications that leverage wetting changes. Figure 7 summarizes the conductivity and water contact angle measurements of the doped P3HT films as a function of doping potential (for the acetonitrile electrolyte). Films doped at potentials below 0.4 V exhibited low electrical conductivity, whereas films doped above 0.7 V developed a wrinkled morphology. Despite these pronounced morphological changes, the conductivity of the highly doped samples remained stable. GIWAXS measurements further confirmed that the microscale structural order was largely preserved, albeit with expansion of the unit cell along the lamellar stacking direction and contraction along the *π*–*π* stacking direction. Notably, films doped within the intermediate potential range of 0.4–0.7 V exhibited both high conductivity and tunable wetting behavior while maintaining only minor morphological changes, indicating that this regime is particularly promising for optimizing functional properties. Accordingly, future research on next-generation multifunctional and responsive electronic systems will primarily focus on this doping window. More broadly, this study highlights how the coupling between electrochemical doping, surface morphology, and wetting behavior can be used to tune interfacial properties in a controlled manner, enabling adaptive and switchable interfaces targeted by external electrochemical stimuli. Given that such phenomena extend to other conducting polymers and mixed ionic–electronic materials subject to coupled charge and structural evolution, electrochemical doping emerges as a powerful, versatile strategy for tailoring interfacial properties.

## Materials and methods

### Materials

ITO-coated glass substrates (thickness 180 nm, CEC0105) were purchased from PGO Präzision Glas & Optic GmbH. P3HT (*M*_*n*_ = 46.6 kg mol^–1^, PDI = 2.4, regioregularity of 95%) was purchased from Merck and used without further purification. 1,2-Dichlorobenzene (DCB), p-Xylene and acetonitrile (ACN) were purchased from Sigma-Aldrich (p.a. grade) and used as received. Tetra-*n*-butylammonium hexafluorophosphate (TBAPF_6_, electrochemical grade) was purchased from Sigma-Aldrich.

### Thin film preparation

P3HT thin films were produced through solution spin-coating from P3HT solutions in 1,2-dichlorobenzene with a concentration of 5 mg ml^−1^, yielding film thicknesses of 10 nm as measured by atomic force microscopy. Prior to deposition, the solutions were stirred overnight at 60 °C ensuring complete P3HT dissolution. The thin films were spin-coated (2500 rpm for 120 s, followed by 3000 rpm for 15 s) on precleaned ITO-coated glass substrates or gold coated substrates (subsequent ultrasonication in water, isopropanol, and acetone). An annealing step (105 °C for 10 min) was performed after spin-coating. Thin film preparation was performed under inert conditions, inside of a glovebox system. After fabrication, the different films were stored over-night under an inert atmosphere (glovebox), ensuring the removal of the remaining solvent. For optimal absorbance in the in situ electrochemical experiment, a thicker film (approximately 60 nm) was prepared by spin-coating a 15 mg ml^−1^ solution at 1000 rpm for 120 s, followed by 3000 rpm for 15 s. Xylene-prepared films were fabricated by spin-coating from P3HT solutions (10 mg ml^−1^ in p-xylene): 1000 rpm for 120 s, followed by 3000 rpm for 15 s, which yielded film thicknesses of 45 nm.

### In situ spectroelectrochemical measurements

P3HT films were electrochemically doped through potentiostatic charging in a three-electrode setup in two different electrolytes: TBAPF_6_ in ACN, and KPF_6_ in an aqueous solution. The reference electrode was an Ag/AgCl wire for the TBAPF_6_/ACN system and a commercial Ag/AgCl electrode for the KPF_6_/H_2_O system. The counter electrode was a platinum ring which allowed the beam to pass through. The absorption spectra of the P3HT films were recorded in real-time by coupling the electrochemical setup with a Zeiss spectrometer system (with MCS611/621 detectors and a CLH600 halogen lamp). After the measurements, all the potentials were calibrated to the value referenced against the redox couple Fc/Fc^+^, added as the internal standard for the ACN electrolyte cells.

### Thin film electrochemical doping

Potentiostatic doping with TBAPF_6_/ACN was performed on different P3HT thin films under an inert atmosphere and within 24 h of their fabrication. This doping process was controlled by a Metrohm PGSTAT101 potentiostat, for a duration of 40 s. Doped P3HT films were stored overnight in the inert atmosphere to completely remove the remaining acetonitrile. The UV–Vis–NIR spectra of the different samples were recorded to characterize the doped state of the P3HT films using the above-mentioned Zeiss spectrometer system. Precleaned ITO-coated glass substrates were used as reference.

### Opto-electronic measurements on 4-line-Au electrodes on glass

To investigate the opto-electronic properties of the doped P3HT thin films, a specially designed substrate featuring four parallel gold electrodes patterned on a glass surface (as shown in Figure S2) was employed. The gold electrodes, 30 nm thick, were deposited on a 3 nm chromium adhesion layer to improve coating quality and film stability. Following film preparation and doping as described above, both optical absorption and electrical conductivity measurements were performed under the inert conditions of a glovebox system. Optical absorption measurements were carried out on the glass region of the modified substrate using the aforementioned Zeiss spectrometer system. Electrical conductivity measurements were performed on the gold regions using a four-point probe configuration coupled to a custom-written LabView program connected to a Keithley 2636 sourcemeter.

### Water contact angle measurements

The water contact angle was measured with a Dataphysis OCA20 instrument coupled with a homemade humidity control system used to regulate relative humidity (r.H.) conditions during the measurement. Before each measurement, the samples were kept inside the chamber at the desired r.H. values for 30 min, allowing the samples to reach equilibrium. For the static water contact angle measurement, a 2 μL deionized water droplet was deposited with an automatic dosing syringe which was controlled by the software SCA [20]. The contact angle was recorded 10 s after the droplet deposition. For the advancing and receding contact angles, an initial 2 μL droplet was deposited on the sample surface. Then, the needle of the syringe was moved into the droplet and water was steadily injected increasing the volume of the drop until 20 μL. During this injecting process, the contact angle increased but the radius of the droplet baseline kept constant until a critical value of the contact angle was reached called advancing contact angle. After this, the radius of the baseline increased while the contact angle remained constant. The receding contact angle was obtained in the reverse process. The obtained data points correspond to the calculated average from 3 measurements, taken for droplets with volumes ranging from 3 to 10 μL. This droplet volume range was chosen as it lies outside the critical contact angle, for which the droplet radius increases linearly with the droplet size [51].

### Morphological characterization

Thin film morphology was characterized using SEM and AFM on samples prepared on ITO substrates. AFM topography images were obtained with a Dimension Icon Atomic Force Microscope from Bruker. SEM imaging was performed with a GeminiSEM 500 from Zeiss, under low voltage conditions (Voltage ≤ 1 kV). For GIWAXS measurements, a gold-coated silicon wafer (1.5 cm × 2 cm) was used as the substrate. GIWAXS measurements were performed at the SAXS/WAXS beamline at the Australian Synchrotron [65]. 15 keV photons were used, with scattering recorded on a Pilatus3-2 M in-vacuum detector. Samples were measured in air, with X-rays only traveling a short distance in air (~ 5 cm), with incident and scattering X-rays otherwise traveling in vacuum. A sample-to-detector distance of ~ 67 cm was used, calibrated with a silver behenate sample. Samples were measured at different incident angles, with the data shown corresponding to the highest scattering intensity taken at an angle just above the critical angle. Data were reduced using a modified version of the Nika [66] implemented in IgorPro.

## Supplementary Information

Below is the link to the electronic supplementary material.

Supplementary file1 (PDF 522 kb)

## Data Availability

The authors declare that all data supporting the findings of this study are provided within the article and its Supplementary Information. Raw data files in alternative formats are available from the corresponding author upon reasonable request.
