# Supplementary material for: Tunable wetting properties of poly(3-hexylthiophene) films with different doping levels
Source: Eur Phys J E Soft Matter. 2026 Jul 17;49(8):61. doi: 10.1140/epje/s10189-026-00608-5 (PMC13379448; doi:10.1140/epje/s10189-026-00608-5)
Supplement: Supplementary file 1 — Supplementary file1 (PDF 522 kb) [file 10189_2026_608_MOESM1_ESM.pdf]

# Supporting Information

# Tunable Wetting Properties of Poly(3-hexylthiophene) Films with Different Doping Levels

Junqi Lu[a], David Neusser[a], David Moser[a], Pedro M. Resende[a,], Chris McNeill[c], Sabine Ludwigs[a]*

[a] *IPOC – Functional Polymers, Institute of Polymer Chemistry (IPOC) University of Stuttgart, Stuttgart 70569, Germany*[c] *Department of Materials Science and Engineering, Monash University, Wellington Road, Clayton, Victoria 3800, Australia*

* Corresponding author: *sabine.ludwigs@ipoc.uni-stuttgart.de*

## 1. ***In-situ*** spectroelectrochemical studies of P3HT films

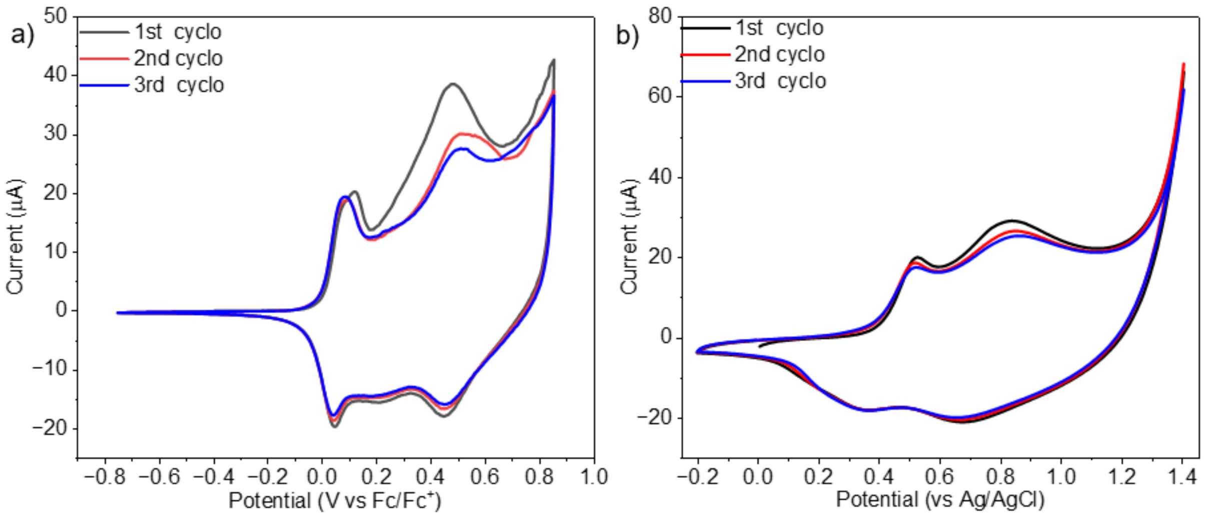


Figure S1. *In-situ* spectroelectrochemical studies of P3HT films prepared from DCB solution. a) CV measured in 0.1 M $TBAPF_6$/MeCN at 20 mV/s on an ITO electrode; 1st to 3rd cycle is shown. b) CV measured in 0.1 M $KPF_6$/$H_2O$ at 20 mV/s on an ITO electrode; 1st to 3rd cycle is shown.

## 2. Opto-electronic properties of electrochemically doped P3HT films in solid state

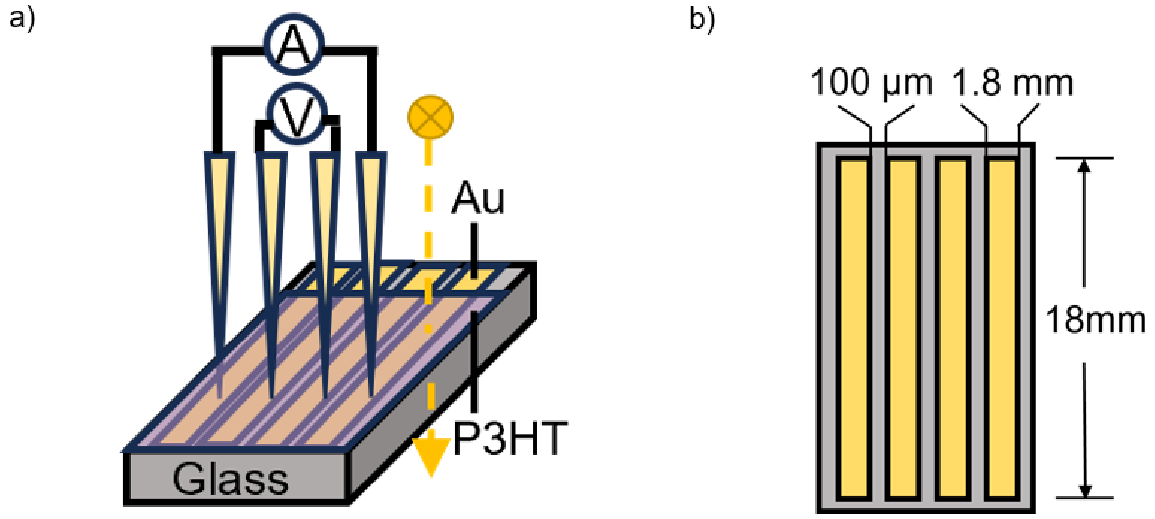


Figure S2. a) Illustration of the measurement of the opto-electronic properties of doped P3HT films prepared from DCB solution on gold-coated substrates. b) Schematic representation of the gold-coated substrate.

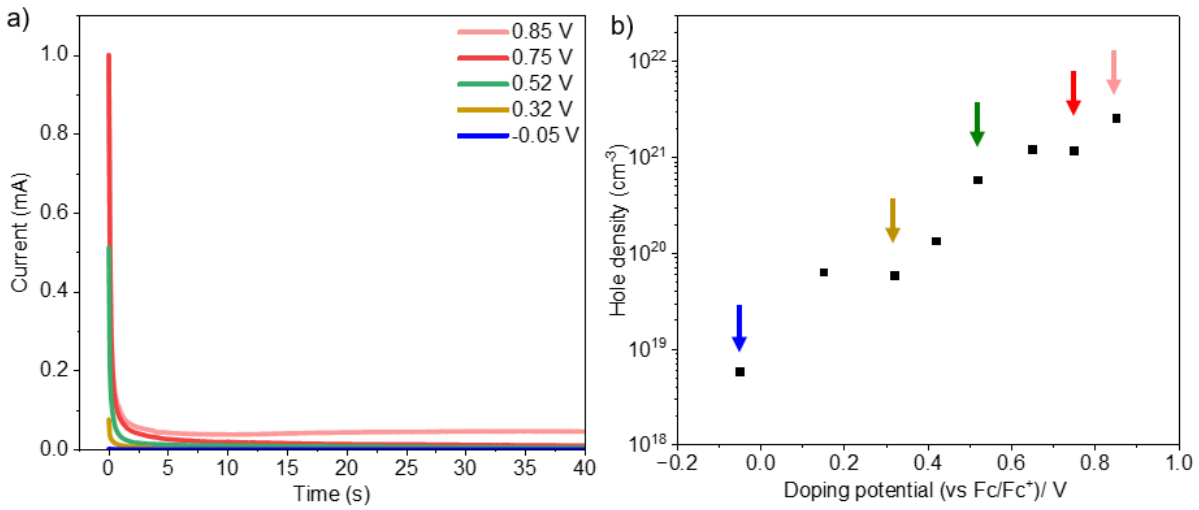


Figure S3. Optical and electronic properties of electrochemically doped P3HT films (a) Chronoamperometric curves recorded during electrochemical doping and (b) corresponding hole density, films obtained by spincoating from DCB solutions on Au-coated glass electrodes.

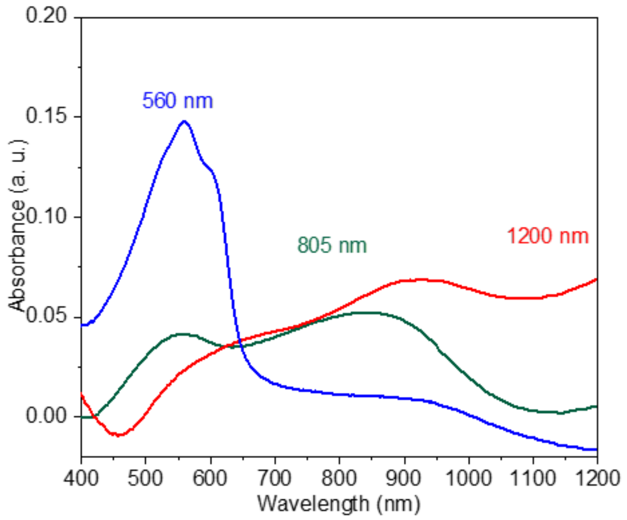


Figure S4: *Ex-situ* solid-state absorption spectra of P3HT films prepared by spin-coating from DCB solutions on ITO electrodes, after doping in 0.1 M $TBAPF_6$/MeCN at 0 V (■), 0.4 V (■), and 0.8 V (■) vs. $Fc/Fc^+$.

**3.Wetting behavior of electrochemically doped P3HT films prepared in organic electrolytes**

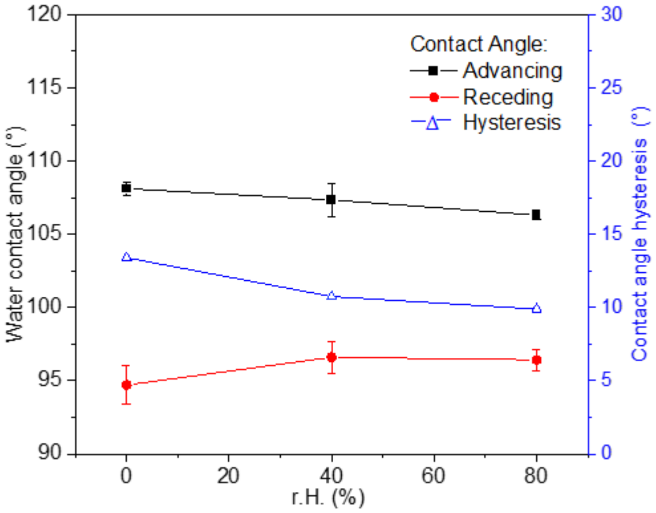


Figure S5. Factors influencing the wetting properties of electrochemically doped P3HT films prepared from DCB solution, as assessed by *ex-situ* solid-state dynamic contact angle measurements, include the measurement conditions (films measured under different relative humidity (r.H.) conditions).

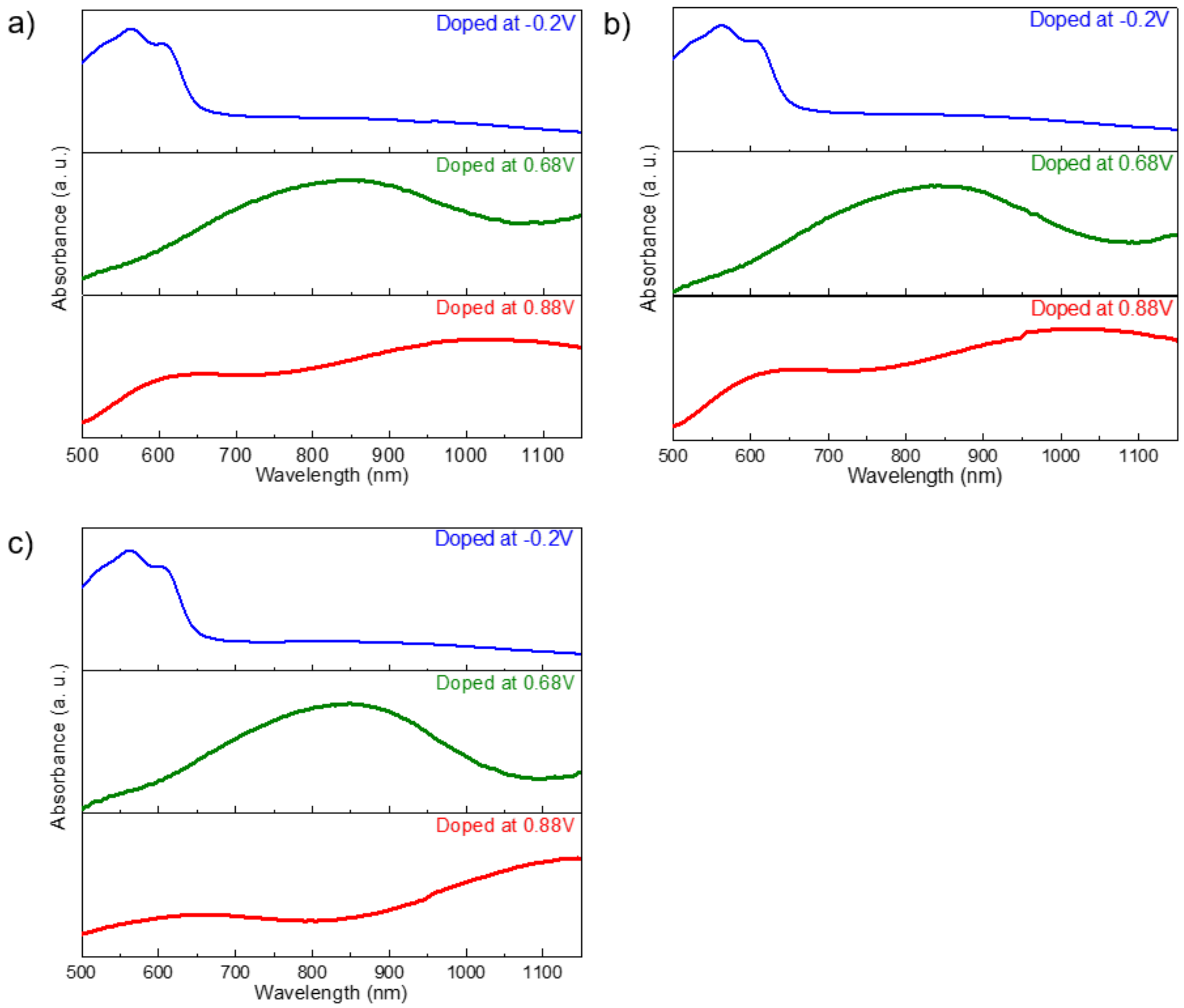


Figure S6. Optical properties of electrochemically doped P3HT films prepared from DCB solution and doped in 0.1M $TBAPF_6$: (a) as-doped state, (b) before water contact angle (WCA) measurement, and (c) after WCA measurement.

## 4. Wetting properties and morphology studies of electrochemically doped P3HT films prepared in aqueous electrolytes

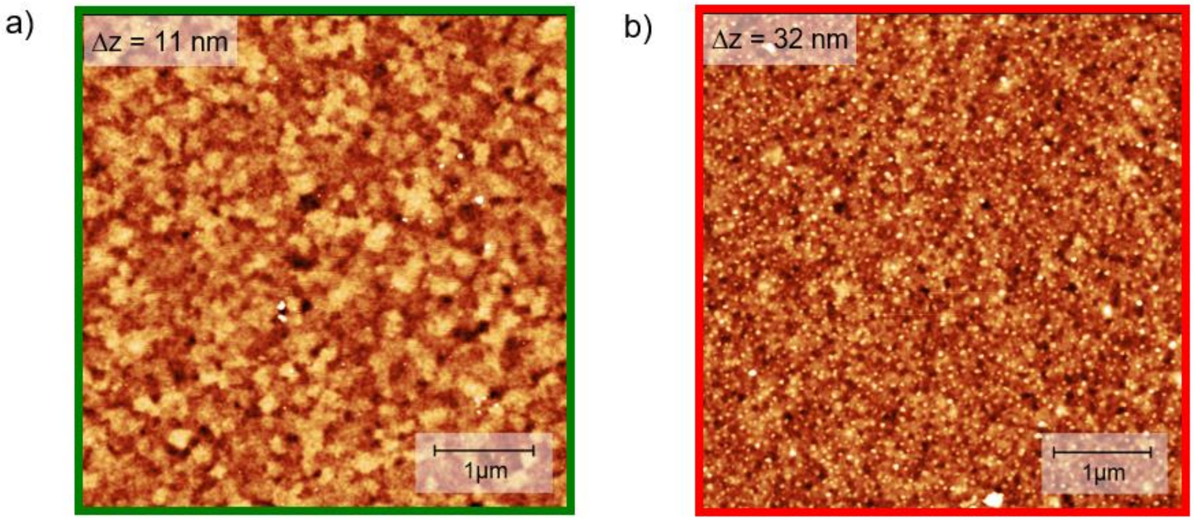


Figure S7. Morphology of electrochemically doped P3HT films prepared from DCB solution. (a) AFM images of films doped at 0.8 V, and (b) 1.2 V vs Ag/AgCl in $KPF_6/H_2O$.

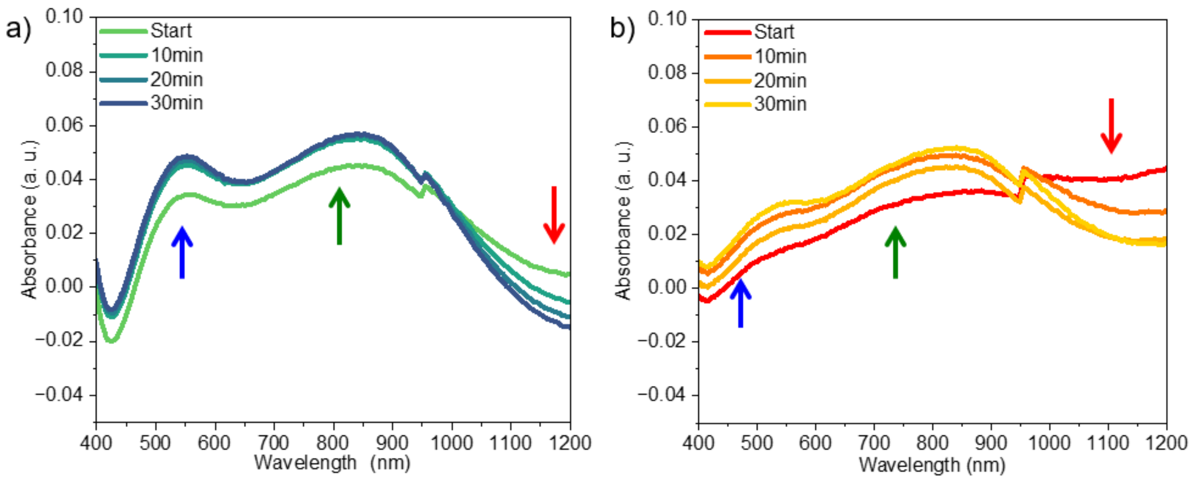


Figure S8. Optical properties of electrochemically doped P3HT films prepared from DCB solution with increasing storage time in inert condition. (a) films doped at 0.8 V, and (b) 1.2 V vs Ag/AgCl in $KPF_6/H_2O$.
